# An Improved InSAR Image Co-Registration Method for Pairs with Relatively Big Distortions or Large Incoherent Areas

**DOI:** 10.3390/s16091519

**Published:** 2016-09-17

**Authors:** Zhenwei Chen, Lei Zhang, Guo Zhang

**Affiliations:** 1 School of Geodesy and Geomatics, Wuhan University, Wuhan 430079, China; guanyuechen@whu.edu.cn; 2 Department of Land Surveying and Geo-Informatics, Hong Kong Polytechnic University, Hong Kong, China; lslzhang@polyu.edu.hk; 3 State Key Laboratory of Information Engineering in Survey, Mapping and Remote Sensing, Wuhan University, Wuhan 430079, China

**Keywords:** InSAR, co-registration, non-translation relation, registration point

## Abstract

Co-registration is one of the most important steps in interferometric synthetic aperture radar (InSAR) data processing. The standard offset-measurement method based on cross-correlating uniformly distributed patches takes no account of specific geometric transformation between images or characteristics of ground scatterers. Hence, it is inefficient and difficult to obtain satisfying co-registration results for image pairs with relatively big distortion or large incoherent areas. Given this, an improved co-registration strategy is proposed in this paper which takes both the geometric features and image content into consideration. Firstly, some geometric transformations including scale, flip, rotation, and shear between images were eliminated based on the geometrical information, and the initial co-registration polynomial was obtained. Then the registration points were automatically detected by integrating the signal-to-clutter-ratio (SCR) thresholds and the amplitude information, and a further co-registration process was performed to refine the polynomial. Several comparison experiments were carried out using 2 TerraSAR-X data from the Hong Kong airport and 21 PALSAR data from the Donghai Bridge. Experiment results demonstrate that the proposed method brings accuracy and efficiency improvements for co-registration and processing abilities in the cases of big distortion between images or large incoherent areas in the images. For most co-registrations, the proposed method can enhance the reliability and applicability of co-registration and thus promote the automation to a higher level.

## 1. Introduction

Interferometric synthetic aperture radar (InSAR) technology has been widely used in acquiring topography maps [1] and detecting surface deformations [2,3]. Co-registration is one of the most important steps in InSAR data processing, as inaccurate co-registrations will reduce the interferometric coherence [4]. In general, the co-registration requires an accuracy up to 1/8 of a pixel to meet the requirement of interferometry [5].

The most commonly used SAR image co-registration method uniformly distributes match patches throughout the imaged area, and then estimates the offsets using the cross-correlation between patches [6]. However, that method has difficulties in obtaining satisfying co-registration results in the following special situations: (1) for the cases of image pairs with relatively big distortions, the match patches may suffer from high uncertainty of the estimated offsets in range or azimuth, and (2) for the cases of image pairs with large incoherent areas, the standard method may lead to a high percentage of error offset estimation and low efficiency. For these two specific situations, we found that the authoritative open sources (such as DORIS, etc.) and commercial software (such as GAMMA, etc.), which are developed based on the standard method, fail to achieve high-accuracy results in range or azimuth. In this paper, we propose a new approach which takes advantage of the prior knowledge of the source of distortions and the characteristics of ground scatterers to address the aforementioned problems. Relating details are described in the following sections.

## 2. Problems with the Standard Co-Registration Methods

There are multiple reasons causing the inapplicability of the standard co-registration method. The well-known reason is the decorrelation of image pairs, primarily caused by long normal baselines, or large deformation between two SAR images. Another reason is the big distortion between the master and slave images. It is known that the transformation relations between two images to be co-registered are usually nonlinear. Hence, the quadric polynomial function in Equation (1) is usually adopted to describe the geometric transformation relationship between the master and the slave images:

(1) $$\left[\begin{array}{c} x_{s}\\ y_{s} \end{array}\right]=f\left(x_{m},y_{m}\right)=\left[\begin{array}{cccccc} a_{0} & a_{1} & a_{2} & a_{3} & a_{4} & a_{5}\\ b_{0} & b_{1} & b_{2} & b_{3} & b_{4} & b_{5} \end{array}\right]\left[\begin{array}{c} 1\\ x_{m}\\ y_{m}\\ x_{m}^{2}\\ y_{m}^{2}\\ x_{m}y_{m} \end{array}\right]$$

where $\left(x_{m},y_{m}\right)$ and $\left(x_{s},y_{s}\right)$ are the coordinates of the master and the slave, respectively, and $\left(a_{0}\cdots a_{5},b_{0}\cdots b_{5}\right)$ are the coefficients of the quadric polynomial function.

**Figure 1:**
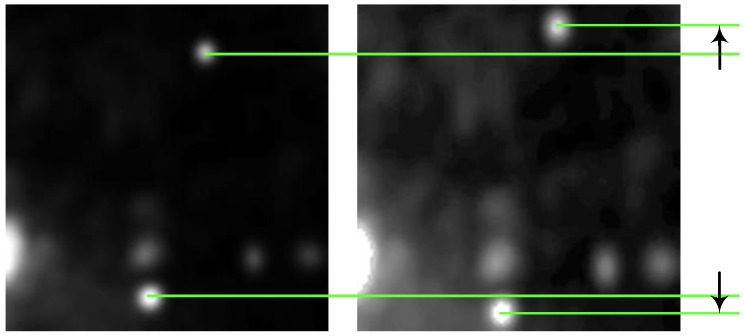
An example of an obvious non-translation relationship between patches. The left and right patches cannot be made to overlap each other, therefore, the relationship between the two patches cannot be described by offset.

The standard method estimates the offsets using cross-correlation between image patches and determines the coefficients in Equation (1) by performing the least square adjustment processing to the offsets. It means that the method is based on the assumption that only a translational relationship exists between each pair of image patches when conducting co-registration. The standard method works when the size of image patches is relatively small (such as 64 × 64 and 128 × 128) and the influence of scale, flip, rotation, and shear (hereafter referred to as non-translation) is little. However, the assumption is unreasonable in some cases where the non-translation relation is too significant to be neglected, as is shown in Figure 1. Because of the non-translation relationship, the image patches in Figure 1 are unable to overlap each other no matter how we move them, making it hard to estimate an accurate offset. In addition, the signal-to-noise ratios (SNR) in this case will be significantly reduced. 

In azimuth, the large offset variations are usually introduced by big angles between orbits. For example, when the scaling rate between patches along the azimuth is 0.01 (it is almost equivalent to $b_{1}$ in Equation (1)), the estimated uncertainty of the offsets in azimuth will be around 0.6 pixel with a 64 × 64 pixel patch. In range, disregarding the topography, the offset variations can be defined as:

(2) $$\frac{\partial off_{rg}}{\partial rg}=\frac{B\cos\left(\theta -\alpha \right)}{R\tan\theta }$$

where $off_{rg}$ is the offset in range, rg is the coordinates in range, B is the normal baseline length, $R_{1}$ and θ are the slope distance and incidence angle corresponding to a pixel in the master, respectively, and α is the angle between normal baseline and horizontal plane. From Equation (2), we know that large offset variations in range are caused by a long baseline. For example, when the vertical baseline is 2000 m, the estimated uncertainty of the offsets in range will be about 0.2–0.3 pixel with the same 64 × 64 pixel patch.

Although we can reduce the patch size to suppress the non-translation between the patches, such an operation will decrease the accuracy of offset estimates as well. Bamler showed that the pixel-offset measuring accuracy of the patch can be determined by

(3) $$\sigma =\sqrt{\frac{3}{2N}}\frac{\sqrt{1-\gamma^{2}}}{\pi \gamma }ovs^{\frac{3}{2}}$$

where N is the number of correlation points of the patch, γ is the coherence, and ovs is the oversampling factor [7,8].

According to Equation (3), too small patch size might lead to inaccurate offset estimates and small SNR values. Such paradox makes the design of a co-registration patch with an appropriate size a challenge when the intersection angle between the satellite orbits or the normal baseline is large.

In addition, as we know, the co-registration accuracy correlates with the patch size which is sometimes manually determined based on personal work experience. In addition, in some cases a satisfying patch size may need multiple pre-tests, greatly hindering the automation of co-registration.

**Figure 2:**
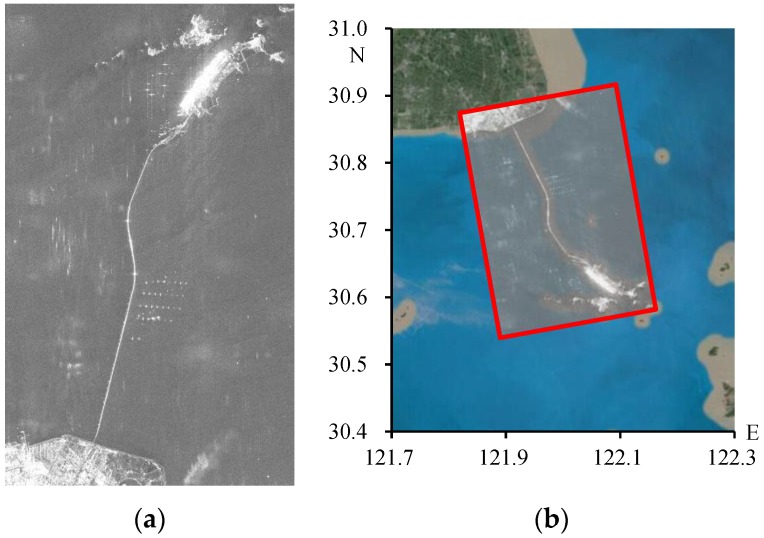
Synthetic aperture radar (SAR) image (**a**) containing large water bodies and its corresponding geographic location (**b**).

Moreover, it is not suitable to use uniformly distributed patches under some circumstances. Take the image in Figure 2 for example, many targets in the image, like the large water area, completely lose coherence. The uniformly distributed patches cause unnecessary work. On the other hand, though most patches with low SNR can be removed by setting thresholds, the rest of the patches with incorrect offset measurements caused by the overestimation effect will significantly reduce the polynomial fitting accuracy [9]. Therefore, how to automatically and reasonably select the effective co-registration area in advance is a problem.

Aiming at solving these problems, we proposed an improved strategy considering both geometrical features and image content. The strategy was tested using 2 TerraSAR-X data from the Hong Kong airport and 21 PALSAR data from the Donghai Bridge. Experimental results indicate that this method can overcome the co-registration difficulties caused by un-paralleling orbits, large normal baseline, and severe decorrelation of signals and effectively improve the co-registration accuracy.

## 3. Proposed Co-Registration Method

**Figure 3:**
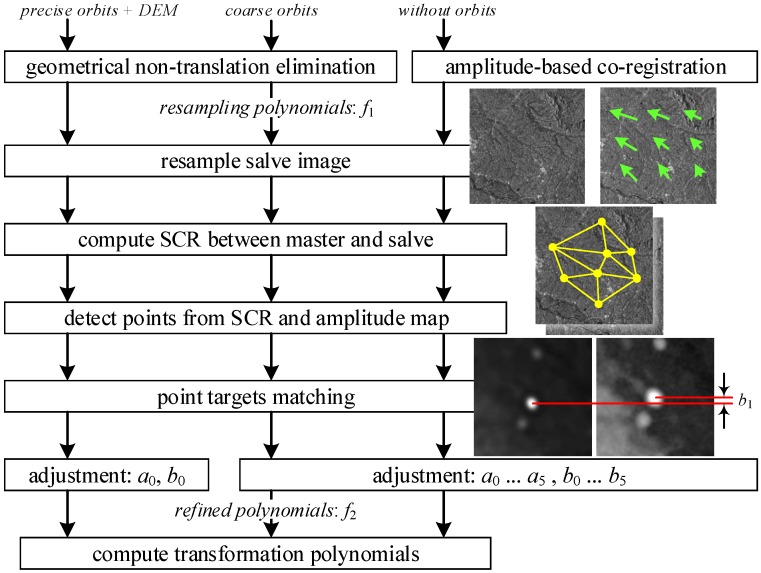
Workflow of the improved co-registration method.

In the proposed co-registration method, both the geometric information and content features are considered to enhance the accuracy and applicability. Specifically speaking, the non-translational transformations between images are first eliminated, and then the pixel co-registration is performed. Finally, the registration points are automatically detected and optimized to further refine the co-registration polynomial. Figure 3 visualizes the principle of the improved co-registration method proposed in the paper.

For the image pair with only coarse orbits, we first define some uniformly distributed points in the master scene, e.g., 8 × 8, and calculate their corresponding locations in the slave scene through the given orbital information. Then, the transformation relation of the master and slave is established by the least square adjustment in Equation (1). Generally, the orbital information and digital elevation model (DEM), if available, are utilized to calculate the space coordinates of each pixel in the master with the Range-Doppler (RD) model. Then, the corresponding image coordinates in the slave can be obtained by inverse computation, and the offset of each pixel in the master and the slave is calculated. Finally, the coefficients in Equation (1) are determined by a series of offsets. The polynomial can be used to eliminate most non-translation caused by big distortion between images in the subsequent steps. Even if the orbital parameters are not entirely accurate or the DEM is not available, the polynomial can reduce the negative influences brought by the non-translation in each patch. Because of the finite precision of orbital parameters, this step can only achieve an accuracy up to pixels. The main significance of the above work is twofold: (1) to provide favourable conditions for more accurate offset estimates in the successive sub-pixel co-registration process, and (2) to facilitate the automatic detection of registration points.

For image pairs with precise orbits, we calculate the offsets pixel by pixel according to the orbital information and related DEM information, and obtain the transformation relationship between images. In this case, the co-registration error is mainly induced by clock drifts of the SAR satellite, which is usually modelled as a constant shift [10,11]. In the following steps, the low-frequency system error will be removed with point targets [12,13].

After the geometric co-registration, it is essential to transform the slave scene into an approximate master geometry. In general, the 2-D sinc interpolation is adopted to perform resampling to the slave scene. Advantages of resampled slave scenes are twofold: One, is it can eliminate the non-translation between image pairs, which is beneficial to get higher SNR and more accurate offset estimates. The other is that it lays the basis for subsequent fast selection of co-registration areas. To enhance the computing efficiency, we store the slave scene in the random access memory (RAM) if the condition of hardware allows.

Though many existing software (such as GAMMA, DORIS, etc.) have employed orbital information to perform co-registration, their purpose is to achieve coarse co-registration, roughly determine the whole translation relations between images, and narrow the search areas for precise co-registration; the non-translation will still affect the precise co-registration. The proposed method eliminates the non-translation relations between images to a certain degree by using orbital parameters, thereby reducing the estimated offset ambiguity of the registration points.

The obtained polynomials are still unable to accurately represent the transformation relationship between the master and the slave. Instead, they show the relation of slave images before and after resampling, which is written as:

(4) $$\left[\begin{array}{c} x_{s}\\ y_{s} \end{array}\right]=f_{1}\left(x_{s^{\prime }},y_{s^{\prime }}\right)$$

where $\left(x_{s},y_{s}\right)$ and $\left(x_{s^{\prime }},y_{s^{\prime }}\right)$ refer to the pixel coordinates of the original slave and the resampled slave, $f_{1}$ is the function in Equation (1).

Then, the automatic detection of registration points is carried out. Serafino cross-correlated a 2-D sinc function template with the SAR image to detect the point targets [14]. The points are detected by searching for local maxima from an ideal 2-D impulse response template and the 2-D cross-correlation surface calculated between oversampled SAR images. However, this method was applied to a single image and did not consider the coherence between images. Moreover, it is of low efficiency, and the detected points might be too densely distributed. Wang et al. detected the registration points from a temporal coherence map, which was roughly obtained from the observed coherence values by dividing them by the geometrical coherence [15]. This strategy works well only when the temporal and normal baselines are small and the master and slave images have been relatively accurately co-registered. Hu et al. simply selected bright pixels using a certain threshold amplitude value [16], which successively avoided incoherent areas (e.g., water bodies). In this paper, we combine the signal-to-clutter-ratio (SCR) and the amplitude information to detect the registration points. To lower the computational cost and reduce the influences of low geometric co-registration precision, the image pairs are firstly subsampled and their SCR are calculated. Then the point targets can be detected by taking pixels with SCR and amplitude values higher than certain thresholds. The automatic detection of the registration points can guarantee more reliable offset estimates for the subsequent co-registration, which makes the co-registration more effective and time-saving, especially for the cases similar to Figure 2. Such operation can avoid major unnecessary computation and greatly improve the efficiency of co-registration.

To avoid points concentration in certain areas (such as cities), detected points are thinned in advance. This means only points with maximum SCR in the areas are reserved. Then the offsets in both the azimuth and range are estimated by cross-correlating pixel patches centred on the rest points. Eliminating non-translation effects in the patches in former steps helps the co-registration. Firstly, a higher SNR and more accurate positions of peak values can be achieved when calculating cross-correlation. Secondly, patches with larger sizes are acceptable while the coherence between images is relatively low. Thirdly, the patch sizes can be automatically assigned by programs without manual work or repetitive tests.

As for images with coarse orbital parameters, the obtained offset estimates are used to fit the coefficients of polynomials in Equation (1), and thus, the accurate transformation relationship between the master and slave can be constructed. In terms of images with precise orbits, the offset estimates are utilized to correct the zero term coefficients $a_{0}$ and $b_{0}$ in the polynomial. Since the errors of clock drifts in azimuth and range of those data during the geometric co-registration are mainly modelled as a constant shift [10,17], not all the coefficients must be calculated. Precise orbits ensure enough accurate co-registration results. For example, an orbit error of 10 cm only introduces a co-registration error of 1/200 pixel [18]. Furthermore, precise orbits contain information of all pixels when conducting geometric co-registration, which makes the non-zero term coefficients more reliable than those corrected by scattered registration points in most cases. Sometimes whether the errors of clock drifts are modelled as a constant shift or not can also be determined by root mean square error (RMSE) when fitting $a_{0}$ and $b_{0}$, and the RMSE should be as small as <0.08 pixel. Otherwise, the previous polynomial should be modified by a linear transformation equation fitted with the offset estimates, or you should perform the same co-registration way as that of the coarse orbital data.

After accurate co-registration, the transformation relationship between master and the first resampled slave is established by simulating Equation (4). The relation can be denoted as:

(5) $$\left[\begin{array}{c} x_{s^{\prime }}\\ y_{s^{\prime }} \end{array}\right]=f_{2}\left(x_{m},y_{m}\right)$$

where $\left(x_{m},y_{m}\right)$ and $\left(x_{s^{\prime }},y_{s^{\prime }}\right)$ refer to the pixel coordinates of the master and the first resampled slave and $f_{2}$ is the function in Equation (4). Combining Equations (4) and (5), the co-registration polynomial of the master and the slave is obtained as follows:

(6) $$\left[\begin{array}{c} x_{s}\\ y_{s} \end{array}\right]=f_{1}\left(f_{2}\left(x_{m},y_{m}\right)\right)$$

In addition, we can resample the slave image onto the grid of the master using Equation (6).

## 4. Experiments and Discussions

In this section, we tested our method using 2 TerraSAR-X data of the Hong Kong airport and the 21 PALSAR data of Donghai Bridge. Multiple comparison experiments were carried out to validate the performance of the proposed method in the cases of (1) great relative distortion between the master and the slave images and (2) large incoherent areas in the images.

### 4.1. Experiment of the TerraSAR-X Data in the Hong Kong Airport

**Figure 4:**
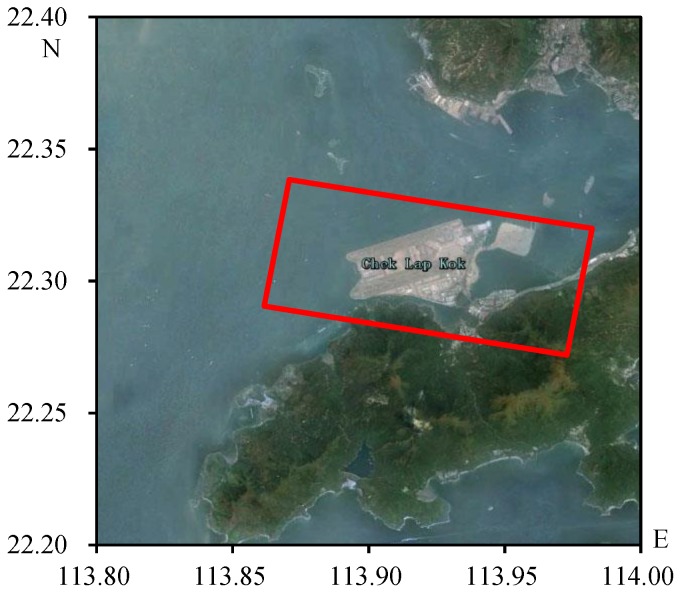
Geographic location of TerraSAR-X data of the Hong Kong airport.

Information of the test TerraSAR-X data of the Hong Kong airport is shown in Table 1 and corresponding geographic location is shown in Figure 4.

**Figure 5:**
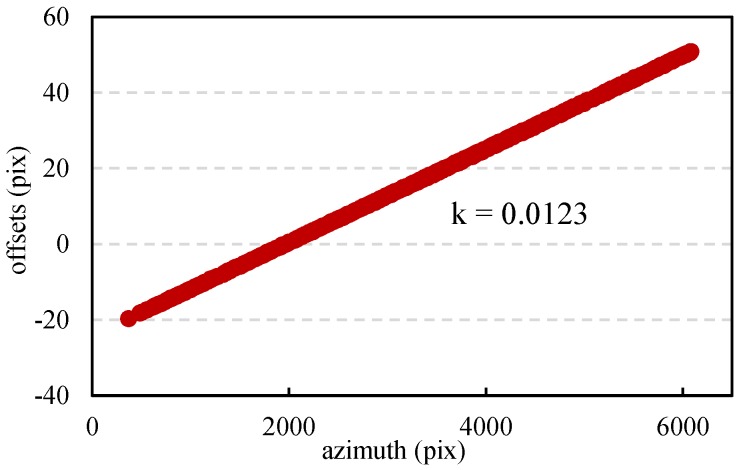
Variation of the offsets along the azimuth.

The orbits intersection angle of the test images is about 6.91 × 10^−6^ rad, resulting in an angle of 0.157 rad in the azimuth. This angle makes the offset estimates vary along the azimuth at a rate of 0.0123, shown in Figure 5. This rate means that the ambiguity of offset estimates in the azimuth of the patch reaches as high as 0.8 pixel, which will influence the accuracy of final co-registration.

To compare our co-registration results with the standard offset measurements, we processed the same datasets to estimate offsets from regularly spaced patches. We uniformly distributed 128 × 128 pixel patches across the images, and estimated the range and azimuth offsets from cross-correlation. To ensure that the match patches were appropriately distributed all over the image, we uniformly distributed 64 patches in range and 48 in azimuth, which lead to 3072 offset estimates.

**Figure 6:**
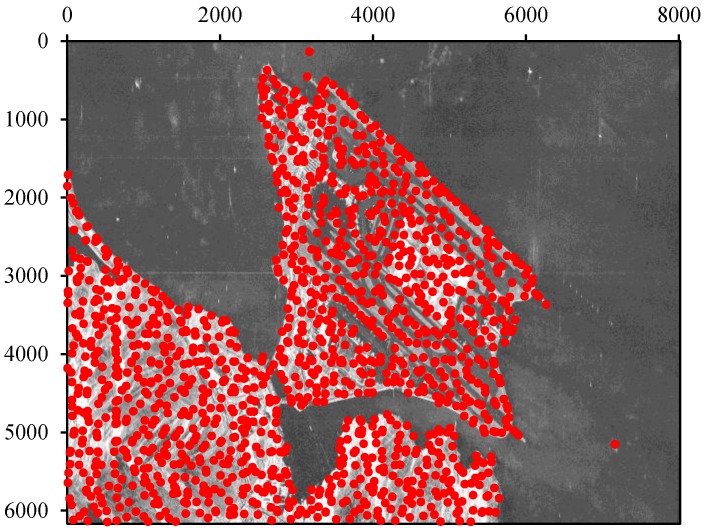
Distribution of the detected registration points by the proposed method.

For the proposed method, the images were co-registered using orbital information, and a series of points were detected with the SCR and amplitude threshold after primary co-registration. On the premise that the point density is comparable to that used in the standard method, we got 1326 registration points, as is shown in Figure 6. The computational cost of our method is as low as about 43% of that of the standard method. This is attributed to the points detection strategy adopted in this paper, which avoids most water areas in the image.

**Figure 7:**
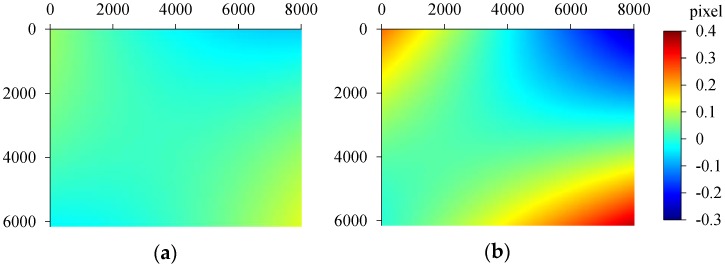
Differences of offset polynomials between the standard method and the proposed method in range (**a**) and azimuth (**b**).

By the standard method, the RMSE values of the quadratic polynomial fitting in azimuth and range are up to 0.064 and 0.269, respectively. However, those values are only 0.029 and 0.051, respectively, with our method which is superior to the standard method. Figure 7 shows the difference of the polynomials using two methods. Obviously, the maximum difference in azimuth exceeds 0.35 pixel.

The low accuracy of the standard method is mainly caused by the intersection angle between images in addition to the influences of a few offset estimates in water areas.

To further verify the functions of the non-translation elimination in co-registration, comparison experiments were performed between the proposed method and the method in literature [14]. Serafino adopted isolated point scatterers (IPS) to do co-registration, which is similar to the strategy based on registration points. However, they did not discuss the non-translation elimination process prior to it. For simplicity, the method based on isolated point scatterers is referred to as the IPS-Based method hereafter. In this method, the point targets are detected using a 2-D sinc function template. The distribution of obtained registration points are too dense, and some points are even on the water area. For better comparison and analysis, we performed the IPS-Based method with the registration points detected by the proposed method.

**Figure 8:**
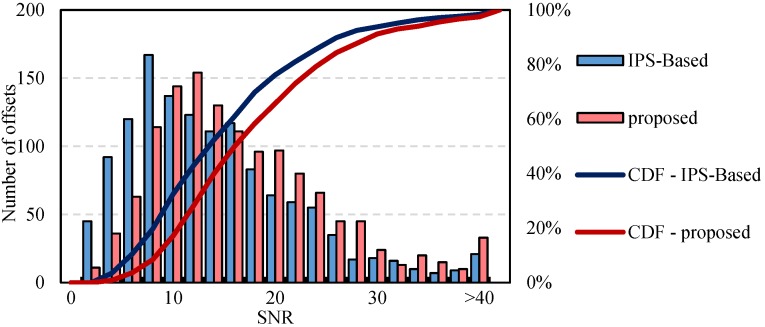
Histogram and cumulative distribution function (CDF) of signal-to-noise ratios (SNR) values.

**Figure 9:**
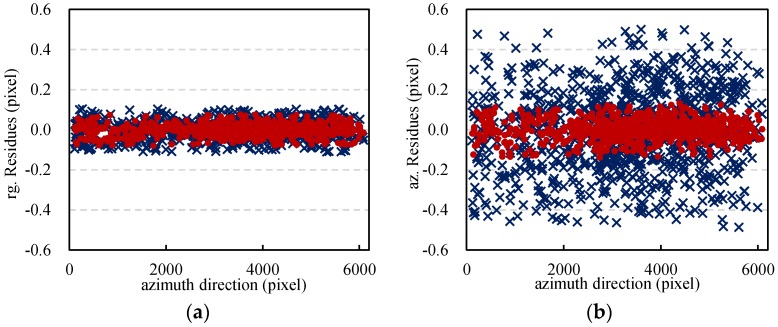
Residues of the offsets in range (**a**) and azimuth (**b**) by the isolated point scatterers (IPS)-Based method and the proposed method. The blue crosses and red points refer to the residues of the offset estimates before and after non-translation elimination.

In Figure 8, the histogram and the cumulative distribution function (CDF) of SNR values are displayed. The SNR values after non-translation elimination are higher than those with non-translation. Then, the polynomials were fitted using the offsets. An iterative least square method was applied to remove the deviation of the offset measurements away from their initial polynomial fit. The residues of the rest offsets in range and azimuth were then calculated, as is shown in Figure 9. For the offset residues in range, similar results were obtained by the two methods. The proposed method achieved a RMSE of 0.0294, a bit smaller than the 0.0430 obtained by the IPS-Based method. While as to the offset residues in azimuth, the residues of the IPS-Based method are very unstable and their RMSE (0.2019) is much greater than the 0.0512 of the proposed method. The analysis of both SNR and residues of offsets in range and azimuth indicates the significant influences of non-translation on the estimation of offsets.

Table 2 lists the results by the standard method, the IPS-Based method, and the proposed method for comparison.

**Figure 10:**
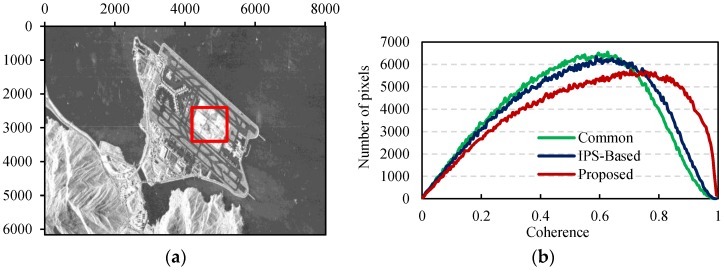
Coherence analysis. (**a**) Land region (red square) for coherence estimation; and (**b**) the coherence histograms by the three methods.

**Figure 11:**
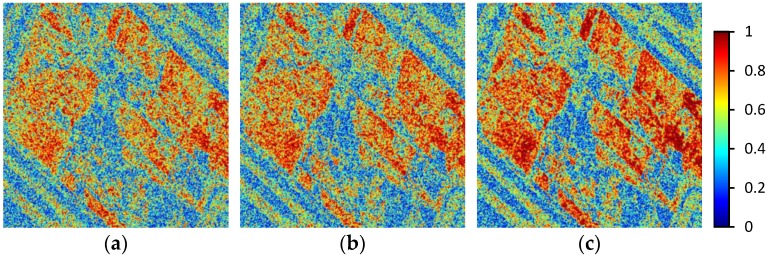
Coherence maps generated by the three methods: (**a**) the standard method; (**b**) the IPS-Based method; (**c**) the proposed method.

The coherence of interferograms is a vital element to evaluate the performance of co-registration [7]. Three interferograms were developed by resampling the slave using the polynomials acquired with the three methods above. To reduce the uncertainties in statistics, a 1000 × 1000 pixel land region containing a large water area was cut out from the original image. The selected region mainly includes buildings, runways, and sand inside the airport, as the red square in Figure 10a shows. We estimated the interferometric coherence from the three interferograms using a Gaussian weighting function. Three developed coherence maps are shown in Figure 11. Figure 10b is the coherence histograms of the three methods. The coherence of the IPS-Based method is slightly higher than that of the standard method, and the proposed method has the highest coherence. The average coherence values of the standard method, IPS-Based method, and our method are 0.509, 0.527 and 0.576, respectively.

### 4.2. Experiment of the PALSAR Data for the Donghai Bridge

**Figure 12:**
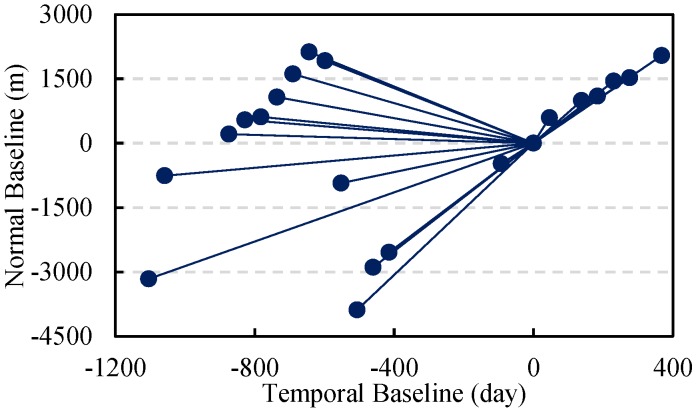
Distribution of the temporal baselines and the normal baselines.

About 32.5 km long, the Donghai Bridge starts from Pudong in Shanghai and ends in Hangzhou Bay. We collected 21 PALSAR images covering the bridge, as the two images in Figure 2 show. The test data span a time duration from January 2007 to January 2011. To ensure a relatively small overall baseline, the data collected on 15 January 2010 were selected as the master and the other data were co-registered to it. The distribution diagram of the normal and temporal baselines of the dataset is shown in Figure 12.

**Figure 13:**
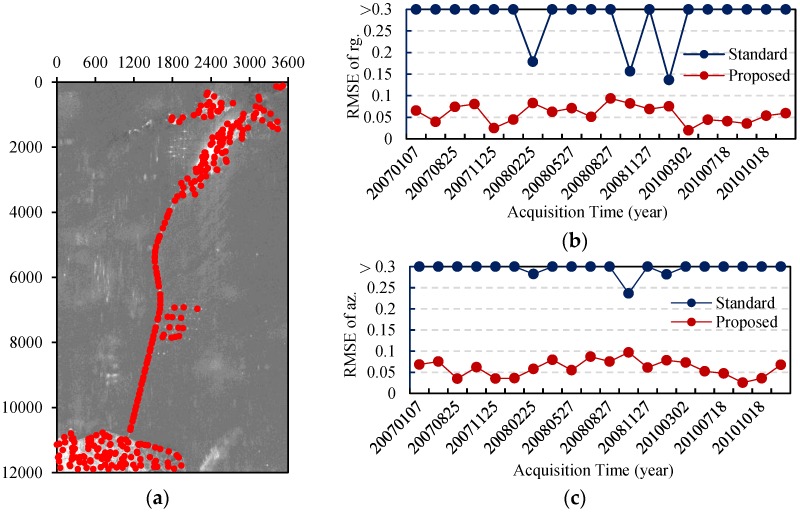
Co-registration results for the Donghai Bridge. (**a**) Distribution of the detected registration points; (**b**) RMSE values of the offset estimates in range; (**c**) RMSE values of the offset estimates in azimuth obtained by both the standard method and the proposed method.

Because water occupies most of the image, the standard method generates many wrong offset estimates, which affects the final polynomial fitting and is time-consuming. However, the proposed method is able to discard the water areas effectively. We obtained 276 registration points with our method and they were distributed as shown in Figure 13a. Figure 13b,c compare the RMSE values of the offset estimates in range and azimuth obtained by both the standard method and the proposed method.

From Figure 13b,c, we can see the standard method cannot satisfy the demands of co-registration of 0.1 pixel in the data set, while the RMSE values in azimuth and range of the proposed method are both within 0.1 pixel. It is worth mentioning that despite the intersection angles of those images, the pairs are not large, the monotonic distribution of the radar backscatters of the bridge regions, and the simple shape of the bridge in patches increase the difficulty of offset estimation. Tiny non-translation will result in inaccurate estimates. Therefore, it is essential to eliminate the non-translation so as to guarantee the co-registration accuracy in the bridge regions.

**Figure 14:**
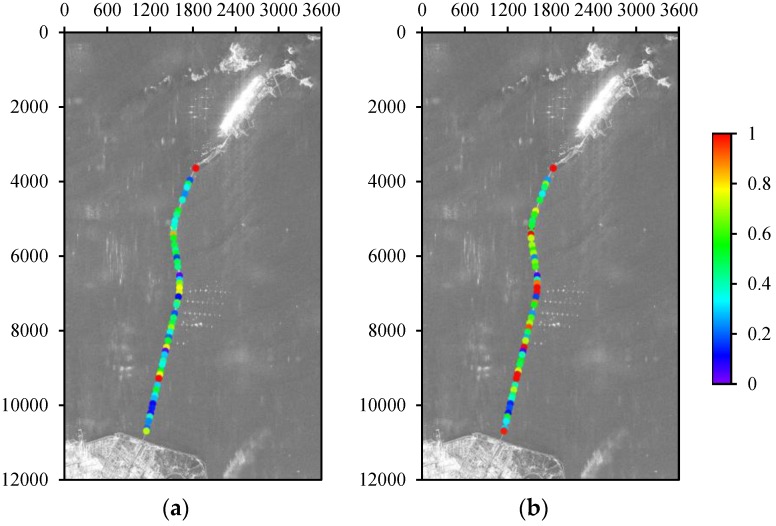
Coherence difference of the bridge between (**a**) the standard method and (**b**) the proposed method.

In this case, the bridge is the most difficult area for co-registration. So we compared and analysed the coherence of the bridge region after co-registration. Because of the errored co-registration results of many image pairs in the stack from the standard method, the interferograms cannot be formed. Hence, an image pair with relatively useful co-registration results is chosen for comparison, which were obtained on 15 January 2010 (master) and 15 October 2009 (slave). The RMSE values in range and azimuth are 0.1365 and 0.2818. We distributed 55 points along the bridge, and estimated the coherence by a 3 × 3 pixel window. Figure 14 shows the coherence difference of the interferograms obtained by the standard and the proposed method. Compared with our method, the standard method has a lower overall interferometric coherence in the bridge regions, which leads to different losses of the interferometric information.

## 5. Conclusions

The standard co-registration method establishes a nonlinear geometric transformation model of the two SAR images with a series of offsets. As discussed in previous sections, this strategy does not work in some situations. On the one hand, significant non-translation relationships between images will be reflected in each patch with a centred registration point, resulting in great ambiguity in the offset estimates. On the other hand, images with abundant incoherent areas will introduce many errored offset estimates. These factors jointly lead to inaccurate image co-registration and reduce the computational efficiency. The basic reason is the standard method does not consider the geometrical features and content, therefore, its reliability and general applicability are affected. To get ideal results using the standard method, multiple tests and accuracy evaluations are usually performed by constantly changing the parameters, such as the size of the co-registration patch. Such a process reduces the automation degree and computation efficiency, and still cannot get satisfying co-registration results.

The main improvement of the proposed InSAR image co-registration method is that we take both the geometrical features and content of the SAR image pairs into consideration, which can be divided into two aspects specifically. (1) The elimination of non-translation. Though many existing methods have employed orbital information to perform co-registration, their objective was to achieve coarse co-registration, roughly determine the whole translation relationships between images, and narrow the search areas for precise co-registration, but the non-translation will still affect the precise co-registration. The proposed method eliminated the non-translation relations between images to a certain degree by using orbital parameters, thereby reducing the estimated offset ambiguity of the registration points. (2) The automatic location of effective co-registration areas. In this paper, the registration points are detected automatically by integrating the information of master and slave. The proposed method is capable of avoiding the decoherent areas effectively compared with the methods using uniformly distributed patches and locating the registration points more quickly and efficiently compared to the other point detection methods. Several experimental results showed that the proposed method can overcome the co-registration difficulties posed to the standard method and effectively improve the accuracy and efficiency of co-registration. In other words, the proposed method improves the reliability and applicability of co-registration. It also reduces the dependence on personal expertise and thus promotes the automation to a higher level.

In terms of the time efficiency of the proposed method, the details are discussed as follows. Our method consists of two stages. In the first stage, the orbital information is used to estimate the rough transformation relation between images. This process is very fast and will not increase the total co-registration time. The second stage is to calculate the offsets by the cross-correlation of patches. Since the improved method can keep some unnecessary co-registration regions out, it costs much less co-registration time than the standard method, especially for images containing many incoherent areas such as water bodies. After the first stage, the first resampling operation should be performed to the slave scene. The 2D sinc interpolator is usually adopted for resampling. Its computational efficiency is much higher than that of offset calculations during co-registration. The reason is that the latter operation involves a Fourier Transform, an inverse transform, a conjugate multiplier, an interpolation to acquire the positions of peaks, and the oversampling in most cases. However, if the resampling results from the first stage must be stored in the hard disk and be read from the disk in the second stage, the consumed time cannot be ignored. Therefore, the resampling results in the first stage are always saved to RAM in practice. With regard to insufficient memory, the images can be divided into sub-blocks to do co-registration. Generally speaking, the proposed method is able to achieve a higher automation degree while taking no more time than the standard method.

## Figures and Tables

**Table 1:** Basic information of TerraSAR-X data of the Hong Kong airport.

	Master	Slave
Acquisition time	29 April 2009	1 June 2009
Wavelength	3.1 cm (X band)	
Incidence angle	38.7°	
Orbit	Descending	
Acquisition mode	SpotLight	
Range resolution	1 m	
Azimuth resolution	1 m	
Range samples	8016	8016
Azimuth samples	6167	6159

**Table 2:** The root mean square error (RMSE) values of the offsets and the calculation amounts of co-registration by the three methods.

	RMSE in Range	RMSE in Azimuth	Num. of Offsets to Estimate
standard	0.064	0.269	3072
IPS-Based	0.043	0.202	1326
Proposed	0.029	0.051	1326
